# Single-Atom Ce-N_4_-C-(OH)_2_ Nanozyme-Catalyzed Cascade Reaction to Alleviate Hyperglycemia

**DOI:** 10.34133/research.0095

**Published:** 2023-03-30

**Authors:** Guangchun Song, Jia Xu, Hong Zhong, Qi Zhang, Xin Wang, Yitong Lin, Scott P. Beckman, Yunbo Luo, Xiaoyun He, Jin-Cheng Li, Kunlun Huang, Nan Cheng

**Affiliations:** ^1^ Beijing Laboratory for Food Quality and Safety, College of Food Science and Nutritional Engineering, China Agricultural University, Beijing 100083, China.; ^2^ School of Mechanical and Materials Engineering, Washington State University, Pullman, WA 99164, USA.; ^3^ Faculty of Chemical Engineering, Yunnan Provincial Key Laboratory of Energy Saving in Phosphorus, Chemical Engineering and New Phosphorus Materials, Kunming University of Science and Technology, Kunming 650000, China.; ^4^ Key Laboratory of Safety Assessment of Genetically Modified Organism (Food Safety), Ministry of Agriculture, Beijing 100083, China.

## Abstract

The enzyme-mimicking catalytic activity of single-atom nanozymes has been widely used in tumor treatment. However, research on alleviating metabolic diseases, such as hyperglycemia, has not been reported. Herein, we found that the single-atom Ce-N_4_-C-(OH)_2_ (SACe-N_4_-C-(OH)_2_) nanozyme promoted glucose absorption in lysosomes, resulting in increased reactive oxygen species production in HepG2 cells. Furthermore, the SACe-N_4_-C-(OH)_2_ nanozyme initiated a cascade reaction involving superoxide dismutase-, oxidase-, catalase-, and peroxidase-like activity to overcome the limitations associated with the substrate and produce •OH, thus improving glucose intolerance and insulin resistance by increasing the phosphorylation of protein kinase B and glycogen synthase kinase 3β, and the expression of glycogen synthase, promoting glycogen synthesis to improve glucose intolerance and insulin resistance in high-fat diet-induced hyperglycemic mice. Altogether, these results demonstrated that the novel nanozyme SACe-N_4_-C-(OH)_2_ alleviated the effects of hyperglycemia without evident toxicity, demonstrating its excellent clinical application potential.

## Introduction

Single-atom nanozymes are nanoparticle catalysts with enzyme-mimicking properties [1–3] and have various advantages, including low cost, good stability, and high catalytic activity [4]. Many studies have shown that single-atom nanozymes are ideal nanozymes [5,6], owing to their geometric structures and maximum atomic utilization [7]. Single-atom nanozymes sites are distributed and have no obvious interaction [8,9], which greatly increases the atomic utilization rate and active center density [10,11]. Moreover, due to their similarities, single-atom nanozyme active sites possess the same catalytic characteristics as natural enzymes [12]. Several single-atom nanozymes have been applied in antisepsis [13], cancer [14], and tumor therapy [15–17] because they can generate reactive oxygen species (ROS) in the tumor environment by mimicking the activity of peroxidase (POD-like) and oxidase (OXD-like) [18,19]. However, an insufficient supply of substrate and H_2_O_2_ in vivo limits the application of single-atom nanozymes in other diseases, such as metabolic diseases.

Metabolic diseases such as obesity and diabetes seriously threaten human health. Diabetes is a highly dangerous metabolic disease, characterized by high blood glucose levels [20,21]. At present, 95% of patients with diabetes in China have type 2 diabetes, which is regarded as one of the largest global health crises facing the world [22,23]. Clinical hypoglycemic drugs mainly include metformin and sulfonylureas [24]. Although these drugs can effectively control the stability of blood glucose in the body, they may cause side effects such as liver damage, weight gain, hypoglycemia, pancreatic degeneration, and gastrointestinal discomfort [25]. Several studies have suggested an association between ROS and glucose metabolism [26–28]. In particular, a Fe_3_O_4_ nanozyme was reported to have potential efficacy in lowering blood glucose by exerting POD-like activity to locally produce •OH, activating adenosine 5'-monophosphate (AMP)-activated protein kinase (AMPK) to improve glucose tolerance and insulin sensitivity [22].

Therefore, this study selected a high-performance single-atom Ce-N_4_-C-(OH)_2_ (SACe-N_4_-C-(OH)_2_) nanozyme with tandem superoxide dismutase (SOD)-, OXD-, catalase (CAT)-, and POD-like activities in liver and muscle glucose-metabolizing tissues to overcome substrate limitations and self-sufficiently produce •OH, thus having a good therapeutic effect in alleviating hyperglycemia.

## Results

### The characterization of the SACe-N_4_-C-(OH)_2_ nanozyme

Our previous studies [29,30] demonstrated that the SACe-N_4_-C-(OH)_2_ nanozyme had high oxygen reduction reaction (ORR) activity and good stability under both alkaline and acidic conditions. The SACe-N_4_-C-(OH)_2_ nanozyme is enriched with single Ce atoms coordinated by N doping and adsorbed hydroxyl species. In this study, we further characterized the structure and composition of the SACe-N_4_-C-(OH)_2_ nanozyme.

**Fig. 1.**
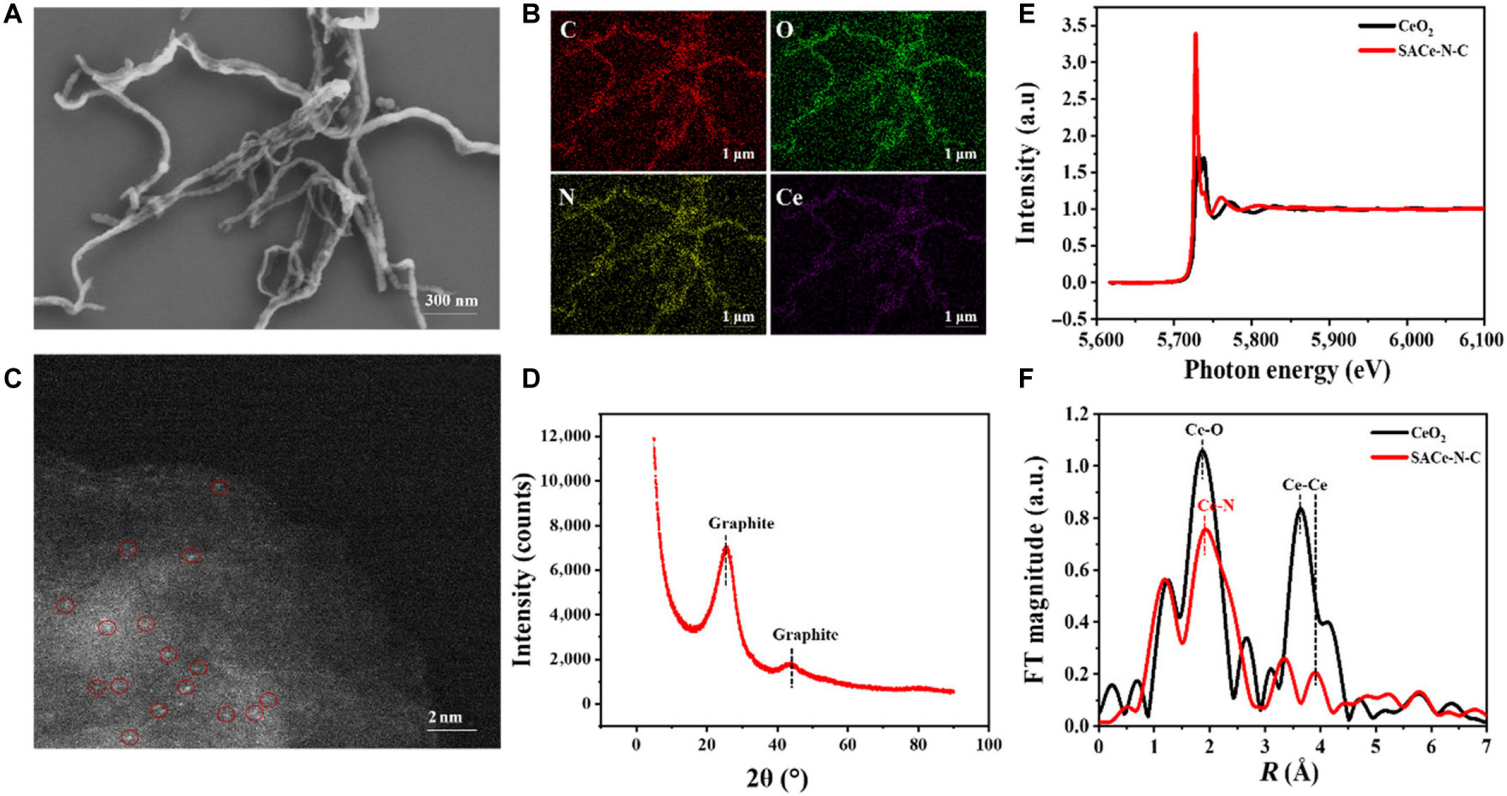
The characterization of the SACe-N_4_-C-(OH)_2_ nanozyme. (A) SEM. (B) The corresponding atomic EDS element mapping of the SACe-N_4_-C-(OH)_2_ nanozyme. (C) A high-magnification DF-STEM enlarged image of the SACe-N_4_-C-(OH)_2_ nanozyme. (D) The XRD of the SACe-N_4_-C-(OH)_2_ nanozyme. (E and F) The XAS of the SACe-N_4_-C-(OH)_2_ nanozymes. a.u., arbitrary units; SEM, scanning electron microscopy; EDS, energy-dispersive x-ray spectroscopy; DF-STEM density functional scanning transmission electron microscope; XAS, x-ray absorption spectroscopy; XRD, x-ray diffraction.

Figure 1A shows the scanning electron microscopy image of the SACe-N_4_-C-(OH)_2_ nanozyme. A SACe-N_4_-C-(OH)_2_ nanozyme with a nanowire structure was observed. The energy-dispersive x-ray spectroscopy element mapping indicated the coexistence of C, N, O, and Ce and a uniform distribution in the SACe-N_4_-C-(OH)_2_ nanozyme (Fig. 1B). As shown in Fig. 1C, abundant isolated bright spots marked by red circles were observed, which could be attributed to single Ce atoms. The x-ray diffraction pattern of the SACe-N_4_-C-(OH)_2_ nanozyme showed 2 characteristic peaks of graphite at 25.2° and 42.5°, suggesting good crystallinity, which is the same as our previous study [29] (Fig. 1D). No crystalline Ce signal was observed, showing that Ce may be present in the SACe-N_4_-C-(OH)_2_ nanozyme as a single-atom species. Figure 1E shows x-ray absorption spectroscopy at the Ce LIII-edge of the SACe-N_4_-C-(OH)_2_ nanozymes and the reference sample of CeO_2_. The black line peak of the CeO_2_ reference is taller than that of the SACe-N_4_-C-(OH)_2_ nanozymes, demonstrating that the Ce oxidation state in the SACe-N_4_-C-(OH)_2_ nanozymes is lower than +4. A relatively weak peak at 3.46 Å was detected in the SACe-N_4_-C-(OH)_2_ nanozymes, which could have originated from a small quantity of nanosized Ce species [29] (Fig. 1F).

### The catalytic mechanism of the SACe-N_4_-C-(OH)_2_ nanozyme

**Fig. 2.**
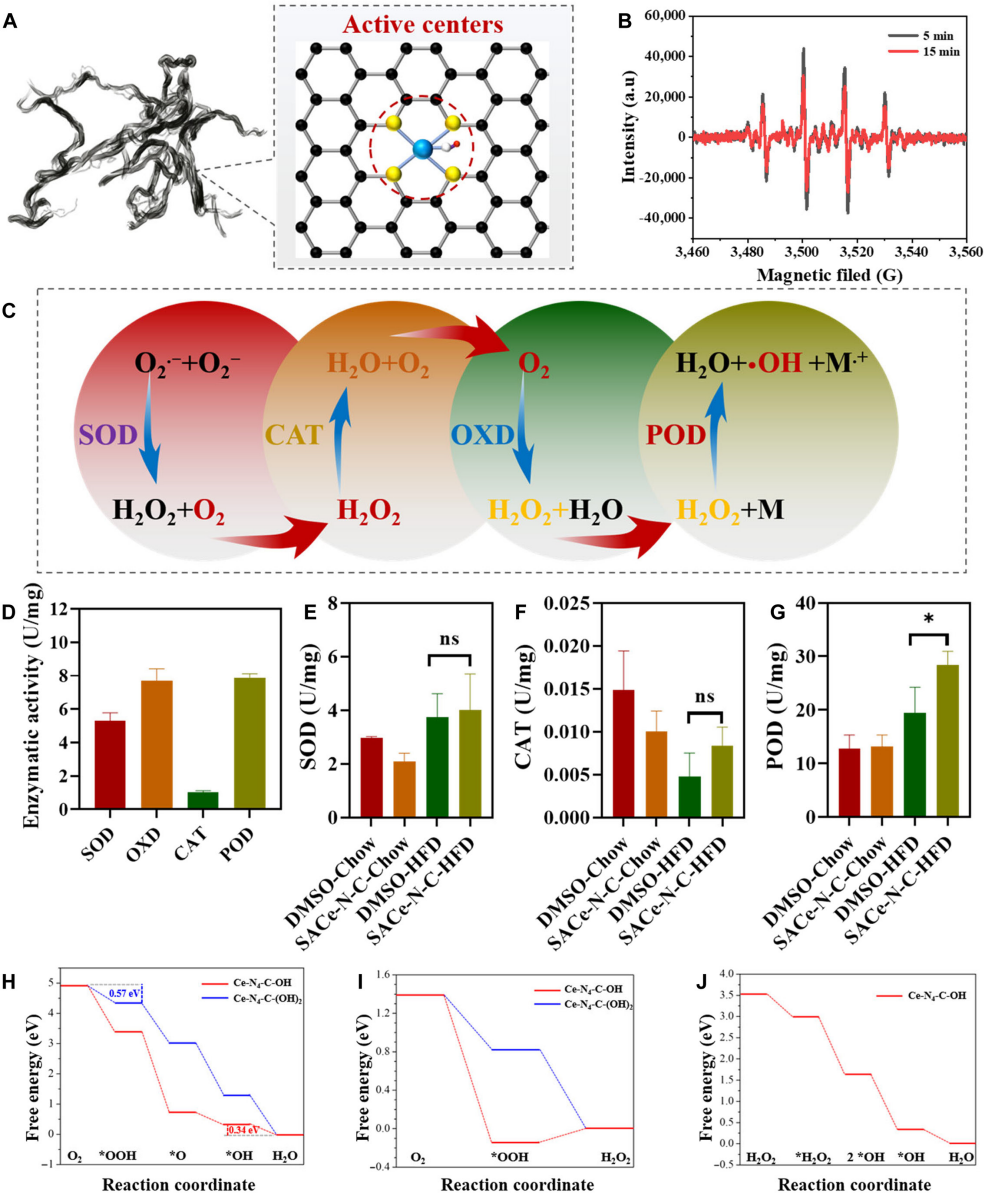
The enzyme-mimicking types and activity of the SACe-N_4_-C-(OH)_2_ nanozyme. (A) The active centers of the SACe-N_4_-C-(OH)_2_ nanozyme. (B) ESR. (C) The POD-like, SOD-like, CAT-like, and OXD-like catalytic mechanism of the SACe-N_4_-C-(OH)_2_ nanozyme. (D) The catalytic activity of the SACe-N_4_-C-(OH)_2_ nanozyme in vitro. (E to G) The SOD-like, CAT-like, and POD-like activity of the SACe-N_4_-C-(OH)_2_ nanozyme in the liver. (H) The free energy diagrams of 4-electron ORR for the Ce-N_4_-C-OH and Ce-N_4_-C-(OH)_2_ models. (I) The free energy diagrams of 2-electron ORR for the Ce-N_4_-C-OH and Ce-N_4_-C-(OH)_2_ models. (J) The free energy diagram of the H_2_O_2_ reduction process for the Ce-N_4_-C-OH model. ESR, electron spin resonance; POD-like, peroxidase-like; SOD-like, superoxide dismutase-like; OXD-like, oxidase-like; CAT-like, catalase-like; ORR, oxygen reduction reaction.

The active center of the SACe-N_4_-C-(OH)_2_ nanozyme is shown in Fig. 2A. A unique single-atom active-site structure coordinated with N-doped, O-doped, and OH-doped carbon materials was observed. We detected ROS using an electron spin resonance instrument at different times and found that more ROS were detected at 5 min (Fig. 2B).

Based on the unique chemical structure of the SACe-N_4_-C-(OH)_2_ nanozyme, we found that it had a variety of enzyme activities, which was more obvious than other M-N-C nanozymes. More specifically, we speculated that the SACe-N_4_-C-(OH)_2_ nanozyme catalyzed SOD-, OXD-, CAT-, and POD-like cascade reactions to produce •OH (Fig. 2C). The POD- and OXD-like activities were higher than the SOD- and CAT-like in vitro (Fig. 2D), indicating that the POD- and OXD-like activities played a vital role in the catalytic process. At the same time, SOD-, OXD-, CAT-, and POD-like activities in the livers of mice were detected by enzyme assay kits; the results are shown in Fig. 2E to G. Clearly, SACe-N_4_-C-(OH)_2_ nanozyme treatment did not significantly affect changes in SOD- and CAT-like activity in mouse livers but significantly enhanced POD- and OXD-like activity. These results proved that the SACe-N_4_-C-(OH)_2_ nanozyme can increase the content of ROS in vivo via POD- and OXD-like activities.

Density functional theory calculations were used to determine the source of the multienzyme-like activity of the SACe-N_4_-C-(OH)_2_ nanozyme. Our previous study [29] demonstrated that Ce-N_4_-C shows significantly strong binding interactions with oxygen-containing intermediates, leading to •OH coverage on the Ce active site owing to a large energy barrier for the reduction of •OH to H_2_O. The strongly bonded •OH species on the Ce active site acted as modifying ligands [31,32], weakening the binding of the intermediates on the catalyst surface. Here, we prepared an •OH species-modified Ce-N_4_-C model (denoted as Ce-N_4_-C-OH, Fig. S1), and the adsorption configurations of intermediates (•OOH, •O, and •OH) on the Ce-N_4_-C-OH model are shown in Fig. S2.

The free energy diagram of the 4-electron ORR for the Ce-N_4_-C-OH model shows that the overpotential-determining step is the reduction of •OH to H_2_O, with a free energy of −0.34 eV (Fig. 2H). The low limiting potential (0.34 V) of the ORR for the Ce-N_4_-C-OH model indicates that the binding interactions between the intermediates and the catalyst surface are still strong, which might result in the partial coverage of •OH on the active site of the Ce-N_4_-C-OH catalyst. Therefore, we constructed an •OH species-modified Ce-N_4_-C-OH model (denoted as Ce-N_4_-C-(OH)_2_, Fig. S1). The adsorption configurations of intermediates (•OOH, •O, and •OH) on the Co-N_4_-C-(OH)_2_ model are shown in Fig. S2. The overpotential-determining step changes from the reduction of •OH to H_2_O for the Ce-N_4_-C-OH model to the formation of •OOH, with a free energy of −0.57 eV, for the Ce-N_4_-C-(OH)_2_ model. Indeed, when the adsorption of intermediates on the catalyst surface is stable, the rate-determining step is the reduction of •OH (e.g., Ce-N_4_-C-OH, Fig. 2H). At the same time, when the adsorption is weak, the reduction of O_2_ to •OOH becomes the rate-determining step (e.g., Ce-N_4_-C-(OH)_2_, Fig. 2H) [33]. The weak adsorption of •OOH on the active site of the Ce-N_4_-C-(OH)_2_ catalyst makes the reduction of •OOH to •O difficult due to poor O-O activation, leading to the selective formation of H_2_O_2_ through the 2-electron ORR process. Moreover, previous studies demonstrated that the 4-electron ORR process dominated when the adsorption-free energy of •OH (ΔG•OH) was close to 0.86 eV, while the 2-electron ORR process led to the competition when ΔG•OH was near 1.02 eV [34]. Our calculated ΔG•OH for the Ce-N_4_-C-(OH)_2_ catalyst was 1.30 eV (Table S1), suggesting that the 2-electron ORR process dominated over the 4-electron ORR process on the Ce-N_4_-C-(OH)_2_ catalyst. Figure 2I shows the free-energy diagrams of the 2-electron ORR for the Ce-N_4_-C-OH and Ce-N_4_-C-(OH)_2_ models. The adsorption configurations of the intermediate (•OOH) for the 2-electron ORR on the Ce-N_4_-C-OH and Ce-N_4_-C-(OH)_2_ models are shown in Fig. S3.

When the H_2_O_2_ molecule was adsorbed on the active site of the Ce-N_4_-C-OH catalyst, it was first cleaved into 2 •OH species that were also adsorbed on the active site. One of the adsorbed •OH molecules subsequently dissociated and desorbed from the catalyst surface, generating active •OH and •OH adsorbed on the active site. The adsorbed •OH reacts with the protonated hydrogen atom under acidic conditions forming •H_2_O species adsorbed on the active site [35]. The catalyst surface returns to its initial state after desorption of the adsorbed •H_2_O species [36–38]. The adsorption configurations of the intermediates (•H_2_O_2_, 2•OH, and •OH) for H_2_O_2_ reduction in the Ce-N_4_-C-OH model are shown in Fig. S4. Figure 2J shows the free energy diagram for H_2_O_2_ reduction on the Ce-N_4_-C-OH catalyst. These reaction steps were downhill in the free energy for H_2_O_2_ reduction, implying facile reactions on the Ce-N_4_-C-OH catalyst. Therefore, active •OH radicals can be generated during H_2_O_2_ reduction. These results indicate that the SACe-N_4_-C-(OH)_2_ nanozyme exhibited excellent catalytic performance.

### The SACe-N_4_-C-(OH)_2_ nanozyme localizes in lysosomes by generating •OH to promote glucose uptake in HepG2 cells

**Fig. 3.**
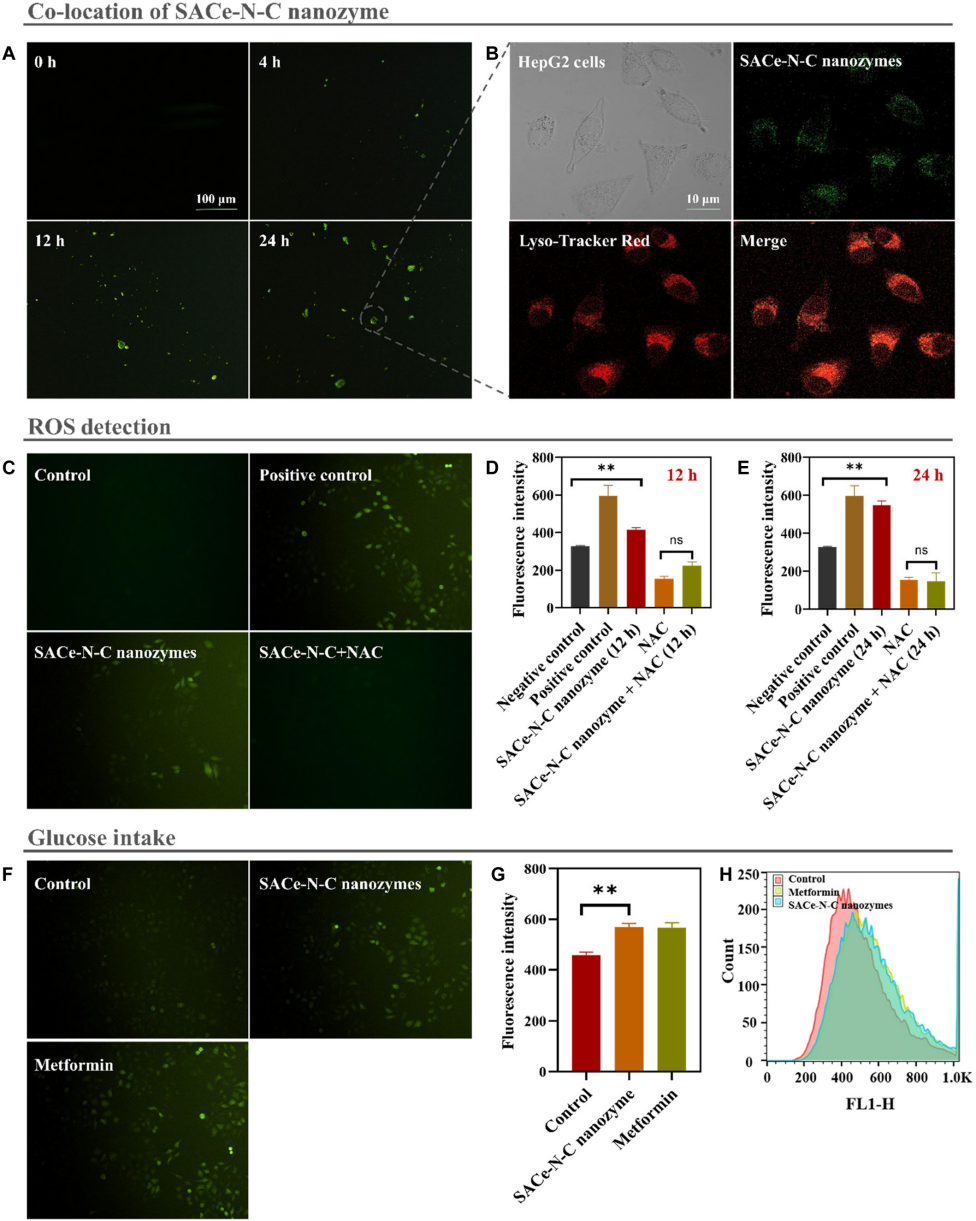
The metabolic responses in HepG2 cells. (A) The intake of the SACe-N_4_-C-(OH)_2_ nanozyme in HepG2 cells at different times. (B) Cell localization (green: SACe-N_4_-C-(OH)_2_ nanozyme-green fluorescent protein, red: Lyso-Tracker Red). (C) The qualitative detection of ROS by an atomic fluorescence microscope. (D) The quantitative detection of ROS by a flow cytometer after 12 h. (E) The quantitative detection of ROS by a flow cytometer after 24 h. (F) The qualitative uptake of fluorescent glucose by an atomic fluorescence microscope. (G) The quantitative uptake of fluorescent glucose by a flow cytometer. (H) Flow cytometry spectroscopy. ROS, reactive oxygen species.

Considering the excellent catalytic performance of the SACe-N_4_-C-(OH)_2_ nanozyme, we hypothesized that it may be a potential therapeutic agent for alleviating the effects of hyperglycemia. First, we explored the cellular uptake of the SACe-N_4_-C-(OH)_2_ nanozyme connected with GFP in HepG2 cells for 4, 12, and 24 h. The results are shown in Fig. 3A. The fluorescence intensity increased with the treatment extension, indicating that the SACe-N_4_-C-(OH)_2_ nanozyme could enter the cells without toxic effects (Fig. S5). Furthermore, the SACe-N_4_-C-(OH)_2_ nanozyme was mainly distributed in acidic lysosomes and exhibited by POD- and OXD-like activities to produce a large amount of •OH (Fig. 3B to E). •OH could not be observed after NAC treatment. At the same time, HepG2 cells treated with the SACe-N_4_-C-(OH)_2_ nanozyme after 12 and 24 h showed significantly enhanced •OH generation ability. The effect was more significant after 24 h of treatment than after 12 h of treatment (Fig. 3D and E).

Next, we explored the effect of the SACe-N_4_-C-(OH)_2_ nanozyme treatment on glucose uptake in HepG2 cells. The results are shown in Fig. 3F. The SACe-N_4_-C-(OH)_2_ nanozyme significantly stimulated the uptake of fluorescent glucose analogs (2-NBDG) in HepG2 cells, and the effect was equivalent to that of metformin at the same concentration. Quantitative analysis by flow cytometry is shown in Fig. 3G and H. These data suggested that the SACe-N4-C-(OH)_2_ nanozyme promoted glucose uptake by producing ROS. Furthermore, the in vitro experiment demonstrated that it mainly distributed in acid lysosomes of HepG2 cells to produce a large amount of •OH through cascade catalytic reaction. Thus, we further explored the effect and molecular mechanism of the SACe-N_4_-C-(OH)_2_ nanozyme in improving glucose metabolism in hyperglycemic mice.

### The SACe-N_4-_C-(OH)_2_ nanozyme is mainly distributed in liver and muscle tissues to alleviate glucose tolerance and insulin resistance in hyperglycemic mice

**Fig. 4.**
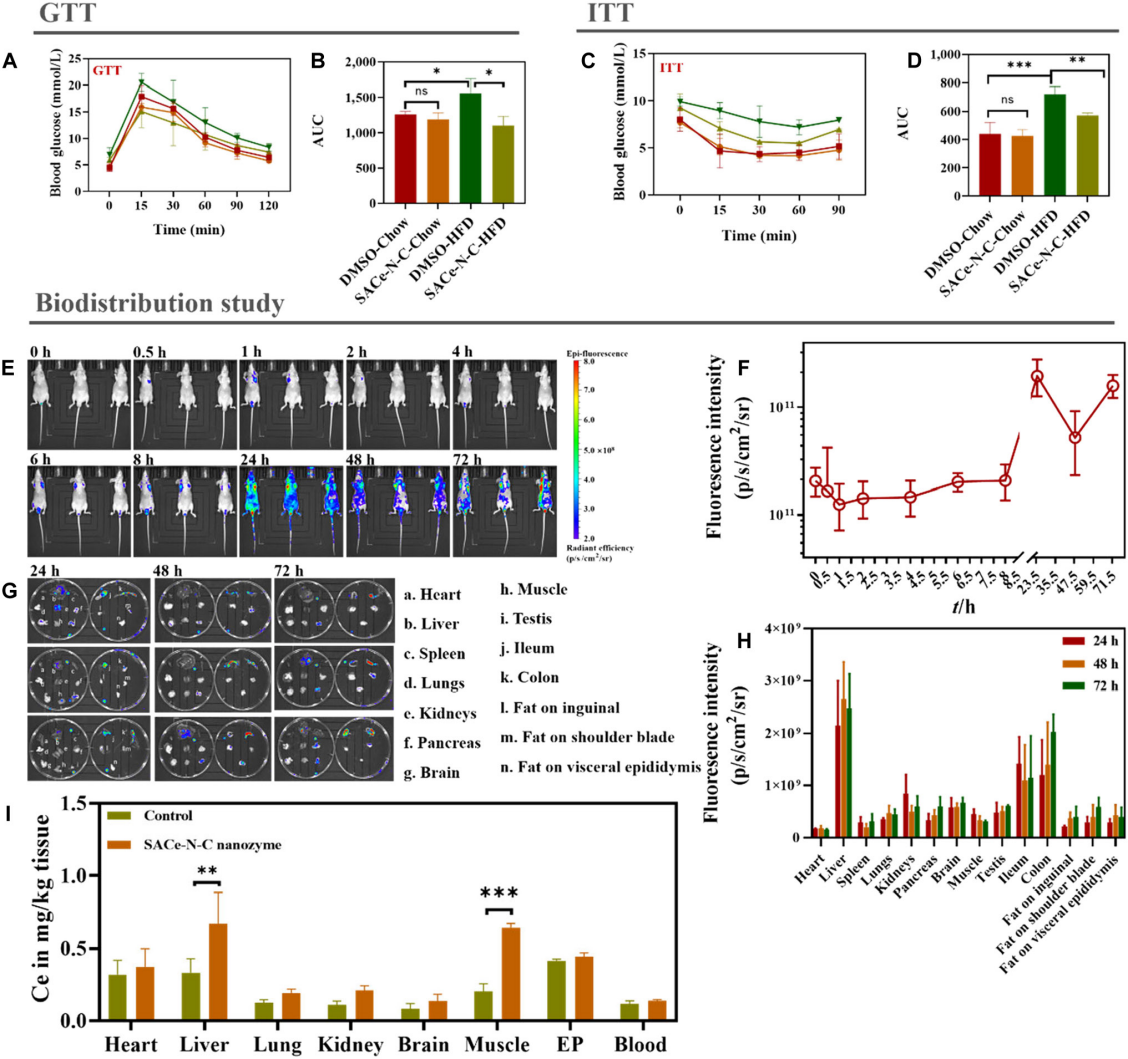
The metabolic responses and distribution in hyperglycemic mice. (A) Glucose tolerance test. (B) Area under the curve of (A) and (C): Insulin tolerance test. (D)  Area under the curve of (C) and (E): The fluorescence of active imaging at different times. (F) The fluorescence quantification of active imaging at a different time. (G) The fluorescence distribution of tissues in mice at 24, 48, and 72 h. (H) The fluorescence quantification of tissues in mice at 24, 48, and 72 h. (I) The biological distribution of Ce in mice.

To evaluate the effect of the SACe-N_4_-C-(OH)_2_ nanozyme on systemic glucose homeostasis, we conducted an oral glucose tolerance test and an insulin tolerance test in hyperglycemic mice fed a high-fat diet (HFD) (Fig. S6). The glucose (Fig. 4A and B) and insulin (Fig. 4C and D) tolerance decreased in the dimethyl sulfoxide (DMSO)-HFD group, and the impairment was ameliorated in the SACe-N_4_-C-(OH)_2_-HFD group. To sum up, these results showed that the SACe-N_4_-C-(OH)_2_ nanozyme alleviated glucose tolerance and insulin resistance in hyperglycemic mice. We also found that the SACe-N_4_-C-(OH)_2_ nanozyme had no obvious effect on the change in body weight of HFD mice (Fig. S7), indicating that the SACe-N_4_-C-(OH)_2_ nanozyme is advantageous for improving glucose metabolism. Therefore, we further explored the distribution of the SACe-N_4_-C-(OH)_2_ nanozyme using in vivo imaging technology to elucidate its mechanism of action.

The highest fluorescence level was detected 24 h after injection, which was consistent with that observed in HepG2 cells (Fig. 4E and F). We also extracted organs at 24, 48, and 72 h after injection and found that among all organs analyzed, the liver had the highest fluorescence level in each period, followed by that in the ileum and colon (Fig. 4H and I). In addition, we evaluated the accumulation of the SACe-N_4_-C-(OH)_2_ nanozyme in each organ tissue after long-term administration by inductively coupled plasma–mass spectrometry (ICP-MS) and found that the SACe-N_4_-C-(OH)_2_ nanozyme was distributed in the liver and muscle tissues (Fig. 4J). Based on these data, we inferred that the SACe-N_4_-C-(OH)_2_ nanozyme alleviated glucose tolerance and insulin resistance mainly by targeting liver and muscle tissues.

### The SACe-N_4_-C-(OH)_2_ nanozyme catalyzed a cascade reaction to produce •OH, increasing the expression of p-Akt, p-GSK3β, and GS

**Fig. 5.**
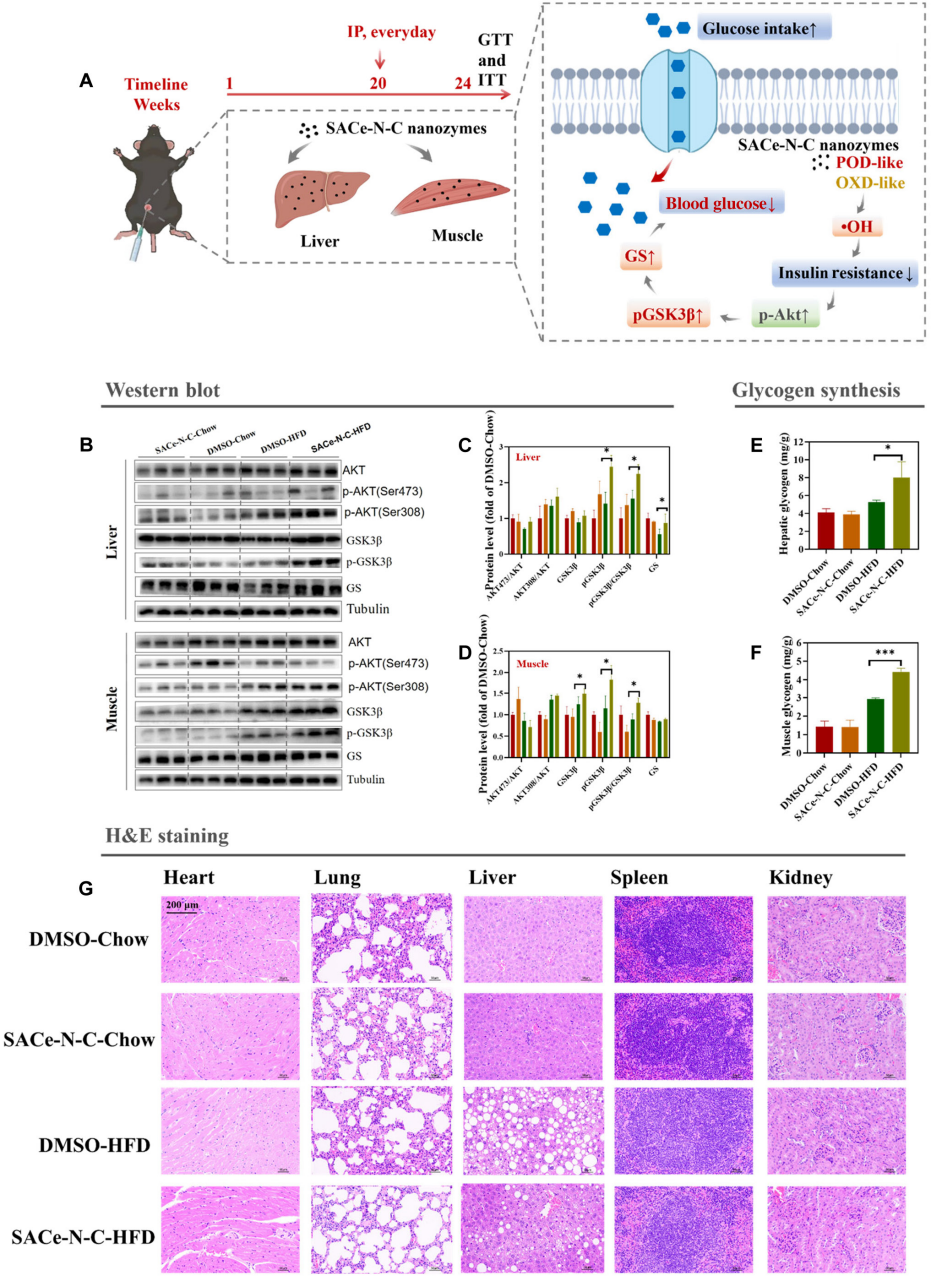
The mechanism and toxicology of the SACe-N_4_-C-(OH)_2_ nanozyme. (A) The metabolic mechanism in vivo. (B) Western blot analysis in liver and muscle tissues. (C) Quantification of Western blot results in the liver. (D) Quantification of Western blot results in muscle. (E) The glycogen synthesis in the liver. (F) The glycogen synthesis in muscle. (G) H&E staining of tissue sections from SACe-N_4_-C-(OH)_2_ nanozyme- or DMSO-treated C57BL/6J mice. Scale bar: 200 μm. DMSO, dimethyl sulfoxide; H&E, hematoxylin and eosin.

The SACe-N_4_-C-(OH)_2_ nanozyme exhibited SOD-, OXD-, CAT-, and POD-like activities to overcome the limitations associated with the substrate and produce •OH, generating local activation of the phosphorylation of protein kinase B (p-Akt), further promoting the phosphorylation of glycogen synthase kinase 3β (p-GSK3β) and the expression of glycogen synthase (GS), thus stimulating glycogen synthesis and improving systemic glucose homeostasis (Fig. 5A).

Previous studies demonstrated that endogenous and exogenous ROS stimulates glucose uptake through a mechanism involving the activation of Akt and/or AMPK [28]. Our results showed that the SACe-N_4_-C-(OH)_2_ nanozyme produced •OH in HepG2 cells (Fig. 3C to E) and liver tissue (Fig. 2E to G). We speculated that this effect was caused by treatment of the SACe-N_4_-C-(OH)_2_ nanozyme, but the role of endogenous POD will need to be determined by further experiments. Therefore, we determined whether the SACe-N_4_-C-(OH)_2_ nanozyme stimulated glucose uptake by activating Akt and/or AMPK. First, we found that the SACe-N_4_-C-(OH)_2_ nanozyme did not affect AMPK protein expression in liver and muscle tissues (Figs. S7 and S8). Interestingly, the protein expression of p-Akt was stronger in the SACe-N_4_-C-(OH)_2_-HFD group than in the DMSO-HFD group (Fig. 5B).

Next, we further explored the expression of the downstream protein AKT. The AKT downstream enzyme GSK3β inhibits glycogen synthesis, promotes gluconeogenesis, and hinders insulin signal transduction. Activated AKT can deactivate GSK3β by phosphorylation (phosphorylation at Ser9), increasing the activity of GS, inhibiting gluconeogenesis, and increasing glycogen production [23,39]. In liver and muscle tissues, the phosphorylation of GSK3β and the expression of GS were significantly increased after treatment with the SACe-N_4_-C-(OH)_2_ nanozyme (Fig. 5B to D). In addition, the SACe-N_4_-C-(OH)_2_ nanozyme significantly enhanced glycogen synthesis in the liver and muscle tissues, enhancing glucose uptake in the blood and lowering blood glucose levels (Fig. 5E and F).

### The toxicology of the SACe-N_4_-C-(OH)_2_ nanozyme in vivo

According to the weight ratio results (Fig. S9), the SACe-N_4_-C-(OH)_2_ nanozyme did not affect weight changes in individual organ tissues. Hematoxylin and eosin (H&E) staining showed no obvious pathological damage in any important organ tissue (Fig. 5G and Fig. S10). Blood chemistry analysis showed no damage to liver biochemistry and function or kidney function (Fig. S11).

These results indicate that the toxicity of the SACe-N_4_-C-(OH)_2_ nanozyme was low or undetectable in hyperglycemic mice. Together, these results suggest that SACe-N_4_-C-(OH)_2_ nanozymes have great potential to alleviate hyperglycemia.

## Discussion

The excellent enzyme-like catalytic performance of the SACe-N_4_-C-(OH)_2_ nanozyme has been studied and applied in previous in vitro experiments [35]. Herein, for the first time, the SACe-N_4_-C-(OH)_2_ nanozyme was applied to alleviate hyperglycemia.

Our study revealed that the SACe-N_4_-C-(OH)_2_ nanozyme was mainly distributed in lysosomes to generate •OH and enhance glucose uptake in HepG2 cells. This effect was similar to that of metformin. We also discovered that the SACe-N_4_-C-(OH)_2_ nanozyme catalyzed a cascade reaction with SOD-, OXD-, CAT-, and POD-like activities to produce •OH in HepG2 cells, demonstrating that the SACe-N_4_-C-(OH)_2_ nanozyme markedly activated the phosphorylation of AKT and promoted the expression of pGSK3β and GS. Furthermore, promoting glycogen synthesis increases glucose intake and lowers blood glucose levels, thus alleviating glucose tolerance and insulin resistance caused by hyperglycemia. However, these cascade reactions need to be proved from multiple enzyme activities of the SACe-N_4_-C-(OH)_2_ nanozyme by successively eliminating various enzyme activities in mice. Therefore, the specific experiments need to be designed and proved in the future.

Overall, our study is the first to demonstrate the role of the SACe-N_4_-C-(OH)_2_ nanozyme in alleviating hyperglycemia and elucidate its mechanism, which may lead to future clinical trials using the SACe-N_4_-C-(OH)_2_ nanozyme to alleviate hyperglycemia.

## Materials and Methods

### Chemical reagents and materials

Dulbecco’s Modified Eagle Medium (DMEM) sugar-free was purchased from Beijing Solarbio Technology Co., Ltd. Metformin was purchased from Sigma-Aldrich (Shanghai) Trading Co., Ltd. The ROS detection kit (DCFH-DA), the nuclear staining kit (dihydrochloride, 4,6-diamino-2-phenyl indole), the lysosomal red fluorescent probe (Lyso-Tracker Red), the POD detection kit, the CAT detection kit, the SOD detection kit, the OXD detection kit, the cell viability detection kit, and the quantitative protein kit were purchased from Shanghai Biyuntian Biotechnology Co., Ltd. The reactive oxygen inhibitor was purchased from Aladdin Reagent Shanghai Co., Ltd.

### The preparation of the SACe-N_4_-C-(OH)_2_ nanozyme

The SACe-N_4_-C-(OH)_2_ nanozyme was prepared based on our previous work [29]. The preparation of the SACe-N_4_-C-(OH)_2_ nanozyme solution was as follows: The obtained SACe-N_4_-C-(OH)_2_ nanozyme was dissolved in a 1/1,000 DMSO solution, and ultrasonic treatment was performed in the cell crusher until it was evenly dispersed for later use.

### The validation of the activity of the SACe-N_4_-C-(OH)_2_ nanozyme

Our previous study showed that the SACe-N_4_-C-(OH)_2_ nanozyme exhibited excellent POD-like activity [35]. In addition, the activities of CAT-, SOD-, and OXD-like proteins were verified using detection kits.

The binding of the SACe-N_4_-C-(OH)_2_ nanozyme and green fluorescent protein was performed as follows:

1. System adjustment. We added 1.0 ml of the SACe-N_4_-C-(OH)_2_ nanozyme solution into a 1.5-ml centrifuge tube and adjusted pH to 8.2 to 8.5 with 0.02 M of K_2_CO_3_ solution.

2. Addition of green fluorescent protein. Then, 5 to 10 μl of green protein (1 mg/ml) was added to the solution (1.0 ml) and shaken at room temperature for 30 min on a multipurpose rotating shaker.

3. Bovine serum albumin (BSA) closure. We added 110 μl of 10% BSA to the above solution, which was shaken at room temperature for 30 min on a multipurpose rotating shaker to seal the area not covered by antibodies on the surface of the particles.

4. Cleaning and purification. The solution was centrifuged at 11,160 × *g* for 20 min, and the supernatant was removed. Subsequently, 1% BSA solution was added and mixed evenly, and the centrifugal cleaning operation was repeated 3 times.

5. Probe resolution. The precipitate was re-dissolved in 100 μl of complex solution (2% BSA+3% sucrose) to obtain a black SACe-N_4_-C-(OH)_2_ nanozyme green fluorescent protein (SACe-N_4_-C-(OH)_2_-GFP) complex.

### The experiment of mice

The animal study was approved by the Animal Ethics Committee of China Agricultural University (approval number: KY 1700025). Animal experiments were performed in the Specific Pathogen-Free Animal Room of the Beijing Agricultural Products Quality Supervision, Inspection, and Testing Center of the Ministry of Agriculture. Six-week-old male C57BL/6J mice were purchased from Beijing Vital River Laboratory Animal Technology Co., Ltd. After an adaptation period of 1 week, mice were divided into 2 groups fed a chow diet (Research Diet, D12450B) and HFD (Research Diet, D12492) for 20 weeks to establish a diet-induced hyperglycemia model [40]. Mice fed a chow diet were randomly divided into 2 groups: a low-fat diet DMSO group (DMSO-Chow) and a low-fat diet SACe-N_4_-C-(OH)_2_ nanozyme group (SACe-N_4_-C-(OH)_2_-Chow). According to the fasting glucose level, mice fed an HFD were randomly divided into 2 groups: the HFD DMSO group (DMSO-HFD) and the HFD SACe-N_4_-C-(OH)_2_ nanozyme group (SACe-N_4_-C-(OH)_2_-HFD). The DMSO-Chow and DMSO-HFD group mice were given 1% DMSO by intraperitoneal injection, and the SACe-N_4_-C-(OH)_2_-Chow and SACe-N_4_-C-(OH)_2_-HFD group mice were administered 10 mg/kg SACe-N_4_-C-(OH)_2_ nanozyme solution (dissolved in 1% DMSO) by intraperitoneal injection for 4 weeks. During treatment, the DMSO-Chow and SACe-N_4_-C-(OH)_2_-Chow groups were fed a chow diet, and each group contained 6 mice. The hyperglycemia model mice, such as the DMSO-HFD and SACe-N_4_-C-(OH)_2_-HFD groups, were fed an HFD, and each group contained 3 mice.

### The SACe-N_4_-C-(OH)_2_ nanozyme entered HepG2 cells over time

For imaging observation by atomic fluorescence microscopy, HepG2 cells (4 × 10^5^ cells/ml) were inoculated in 6-well petri dishes at 37 °C and 5% CO_2_ for 24 h, and then treated with SACe-N_4_-C-(OH)_2_-GPF for 4, 12, and 24 h, respectively.

### Co-location of the SACe-N_4_-C-(OH)_2_ nanozyme

HepG2 cells were inoculated in a confocal culture dish at 37 °C and 5% CO_2_ for 24 h, then treated with the SACe-N_4_-C-(OH)_2_ nanozyme for 24 h, and washed 3 times with phosphate-buffered saline (PBS). Then, the Lyso-Tracker Red raw solution was dissolved in DMEM (volume ratio: 1:13.33). Lyso-Tracker Red solution (300 μl) was added to each petri dish to stain the cell membrane for 60 min and then washed 3 times with PBS. Next, cells were fixed with 300 μl of 4% paraformaldehyde, treated at room temperature for 20 min, and washed 3 times with PBS. Finally, 500 μl of PBS was added for confocal imaging.

### The effect of the SACe-N_4_-C-(OH)_2_ nanozyme on ROS in HepG2 cells

HepG2 cells (4 × 10^5^ cells/ml) were inoculated in a 6-well petri dish and incubated for 24 h. The SACe-N_4_-C-(OH)_2_ nanozyme and SACe-N_4_-C-(OH)_2_ nanozyme + reactive oxygen inhibitor (NAC, 5 mmol/L) were dissolved in DMEM. After 24 h of treatment, the cells were treated with 20 μmol/L DCFH-DA for 45 min to detect intracellular ROS levels. Then, cells were washed 3 times with PBS. Imaging was performed using a TCS SP8 confocal microscope. ROS were quantified using flow cytometry.

### The effect of the SACe-N_4_-C-(OH)_2_ nanozyme on glucose uptake in HepG2 cells

HepG2 cells (4 × 10^5^ cell/ml) were inoculated in 6-well petri dishes for 24 h and treated with the SACe-N_4_-C-(OH)_2_ nanozyme, metformin (200 mg/kg), and their mixture for 24 h. The cells were washed 3 times with PBS, and different concentrations of fluorescent glucose analogs (2-NDBG, 0, 100, and 200 μmol/L) were dissolved in sugar-free DMEM. The cells were treated for 30 min and then washed 3 times with PBS. A confocal laser microscope was used for imaging, and the fluorescence content was measured using a 96-well plate fluorescence spectrophotometer at 540 nm for quantification.

### The assay of glucose tolerance test

C57B/6L mice were tested at week 23 using a glucose concentration of 2.0 g/kg body weight. The specific operation steps are as follows:

1. Mice were fasted for 12 h, 1 day before the experiment, but normal drinking water conditions were ensured.

2. On the second day, the mice were weighed and labeled, and approximately 1 mm was cut off at the end of the tail with sterilized scissors, and a drop of blood was gently squeezed out along the tail to dry it with paper towels. The second drop of blood was squeezed out. A glucose meter was used to measure the fasting blood glucose of the mice, recorded as the 0-min blood glucose value.

3. The corresponding volume of glucose solution was injected into the abdominal cavity of the mice according to the recorded body weight, which was recorded as 0 s.

4. The corresponding blood glucose values at 15, 30, 60, 90, and 120 min were measured, and the physiological status of the mice was observed at any time during the entire process.

5. After the experiment, the mouse tail was wiped gently with cotton alcohol, and food and water were provided.

### The assay of insulin resistance test

C57B/6L mice were tested for insulin resistance at week 23 using an insulin concentration of 0.75 U/kg body weight. Glucose tolerance and insulin tolerance were tested 1 day later. The specific operation steps were as follows:

1. Mice were fasted for 4 h, but normal drinking water conditions were maintained.

2. After weighing and marking the mice, sterilized scissors were used to cut off approximately 1 mm from the end of the tail of the mice, gently squeeze out a drop of blood along the tail that was dried with paper towels, and then squeeze out the second drop of blood. A glucose meter was used to measure the fasting blood glucose of the mice, recorded as the 0-min blood glucose value.

3. The corresponding volume of insulin solution was injected into the abdominal cavity of the mice according to the recorded body weight, which was recorded at 0 s.

4. The corresponding blood glucose values at 15, 30, 60, and 90 min were measured, and the physiological status of the mice was observed during the entire process.

5. After the experiment, the mouse tail was wiped gently with cotton alcohol, and food and water were provided.

### Biodistribution study

#### ICP-MS

Ten mouse organs—liver, muscle, brain, heart, spleen, kidney, epididymis (EP), pancreas, lung, and heart—were collected to study the biological distribution of the SACe-N_4_-C-(OH)_2_ nanozyme. The samples of each group were mixed with digestive solution (HNO_3_+HCl+HClO_4_, volume ratio: 3:1:2) and heated to 100 °C, and the Ce content in tissues and organs was measured by ICP-MS.

#### In vivo imaging

Nine mice were intraperitoneally injected with SAC-N-C-GFP, and a Lumina II imaging system was used for imaging at 0, 0.5, 1, 2, 4, 6, 8, 18, 24, 48, and 72 h after injection. The mice were sacrificed at 24, 48, and 72 h after imaging [41], and the inguinal fat, back brown fat, epididymal fat, subcutaneous fat, heart, liver, spleen, lung, kidney, pancreas, brain, muscle, testis, ileum, and colon were collected for imaging and quantitative calculation [42].

### Blood panel analysis and blood biochemistry

The collected blood was cleared for hematological index testing. Alanine aminotransferase, aspartate aminotransferase, uric acid, alkaline phosphatase, creatinine, blood urea nitrogen, cholesterol, triglyceride, high-density lipoprotein, and low-density lipoprotein levels were measured by blood biochemistry [43].

### Weight ratio

After fasting for 12 h, blood was collected, and the mice were sacrificed. The following organs were dissected, separated, and weighed: heart, liver, spleen, lung, kidney, brain, subcutaneous fat, EP, and testicles; organ coefficients were calculated. Part of the organs was fixed in 4% paraformaldehyde and another part was frozen at –80 °C.

### H&E staining

The mouse heart, liver, spleen, lungs, kidneys, subcutaneous fat, the pancreas, testis, EP, ileum, and colon viscera were fixed in 4% paraformaldehyde solution, dehydrated, paraffin embedded, sectioned, and stained with H&E, and then during imaging, they were histopathologically observed under a light microscope.

## Data Availability

The data that support the findings of this study are available from the corresponding author upon reasonable request.
