# Supplementary material for: Single-Atom Ce-N4-C-(OH)2 Nanozyme-Catalyzed Cascade Reaction to Alleviate Hyperglycemia
Source: Research (Wash D C). 2023 Mar 30;6:0095. doi: 10.34133/research.0095 (PMC10062498; doi:10.34133/research.0095)
Supplement: Supplementary 1 — Figs. S1 to S11 Table S1 [file research.0095.f1.docx]

Single-atom Ce-N_4_-C-(OH)_2_ Nanozyme-Catalyzed Cascade Reaction to Alleviate Hyperglycemia

Guangchun Song^1&^, Jia Xu^1&^, Hong Zhong^2^, Qi Zhang^1^, Scott P. Beckman^2^, Yunbo Luo^1^, Xiaoyun He^1^, Jin-Cheng Li^3*^, Kunlun Huang^1,4*^, Nan Cheng^1*^

^1^Beijing Laboratory for Food Quality and Safety, College of Food Science and Nutritional Engineering, China Agricultural University, Beijing 100083, China

^2^School of Mechanical and Materials Engineering, Washington State University, Pullman, WA 99164, USA

^3^ Faculty of Chemical Engineering, Yunnan Provincial Key Laboratory of Energy Saving in Phosphorus, Chemical Engineering and New Phosphorus Materials, Kunming University of Science and Technology, Kunming 650000, China

^4^ Key Laboratory of Safety Assessment of Genetically Modified Organism (Food Safety), Ministry of Agriculture, Beijing 100083, China

E-mail: [jinchengli@kust.edu.cn](mailto:jinchengli@kust.edu.cn); [hkl009@163.com](mailto:hkl009@163.com); [chengnanFSNE@cau.edu.cn](mailto:chengnanFSNE@cau.edu.cn)


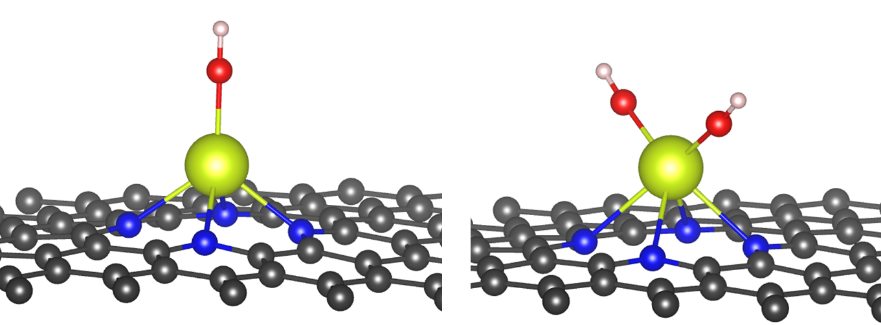


**Figure S1**. The structures of Ce-N_4_-C-OH (left) and Ce-N_4_-C-(OH)_2_ (right) models. Gray, blue, red, white, and yellow balls represent C, N, O, H, and Ce atoms, respectively.


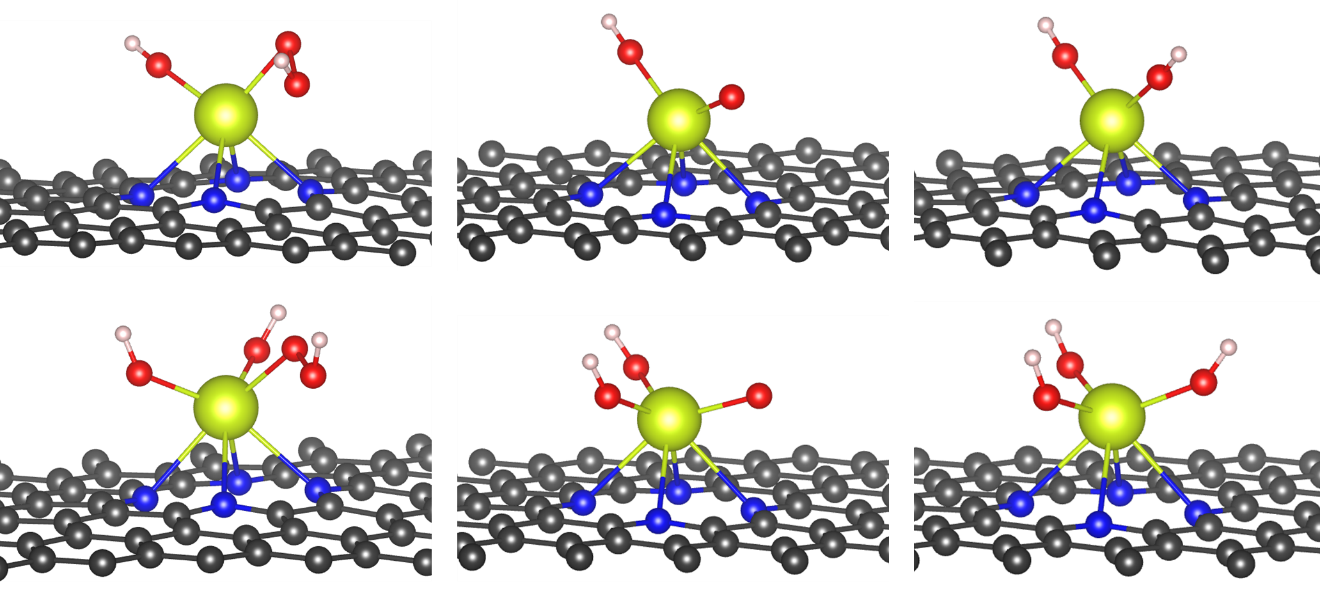


**Figure S2**. The adsorption configurations of oxygen-containing intermediates (*OOH, *O, and *OH) on Ce-N_4_-C-OH (up) and Ce-N_4_-C-(OH)_2_ (down) models for four-electron ORR process. Gray, blue, red, white, and yellow balls represent C, N, O, H, and Ce atoms, respectively.


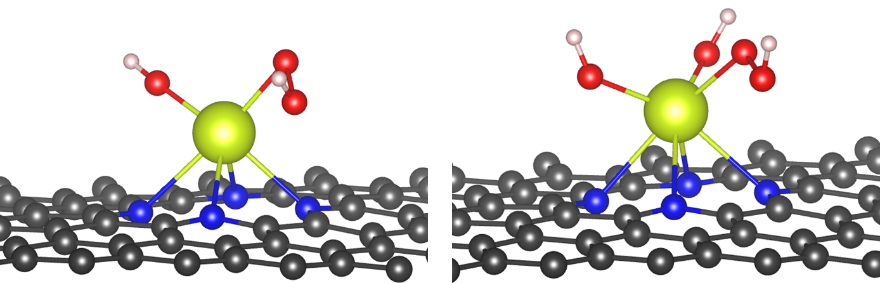


**Figure S3.** The adsorption configuration of oxygen-containing intermediate (*OOH) on Ce-N_4_-C-OH (left) and Ce-N_4_-C-(OH)_2_ (right) models for two-electron ORR process. Gray, blue, red, white, and yellow balls represent C, N, O, H, and Ce atoms, respectively.


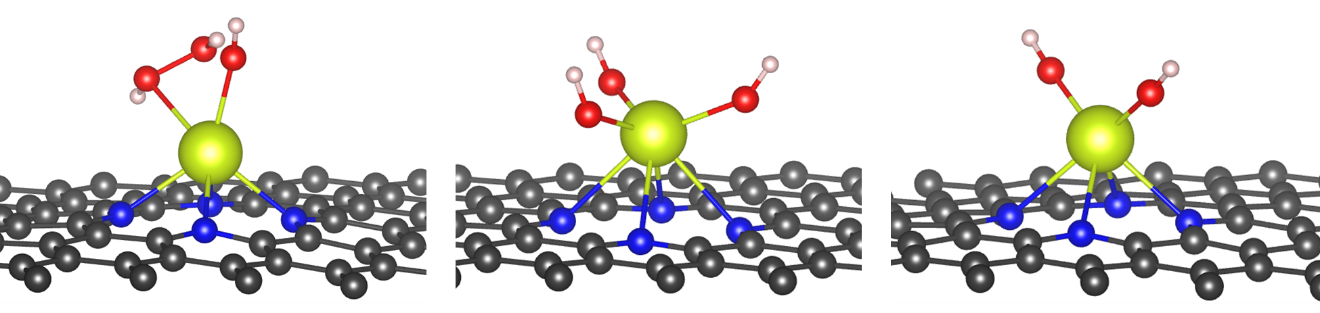


**Figure S4.** The adsorption configurations of oxygen-containing intermediates (*H_2_O_2_, 2*OH, and *OH) on Ce-N_4_-C-OH model for H_2_O_2_ reduction process. Gray, blue, red, white, and yellow balls represent C, N, O, H, and Ce atoms, respectively.

**Table S1** Adsorption free energy (eV) of ORR intermediates on catalyst surface.

| **Model** | *OOH | *O | *OH |
| --- | --- | --- | --- |
| Ce-N_4_-C-OH | 3.39 | 0.73 | 0.34 |
| Ce-N_4_-C-(OH)_2_ | 4.35 | 3.01 | 1.30 |


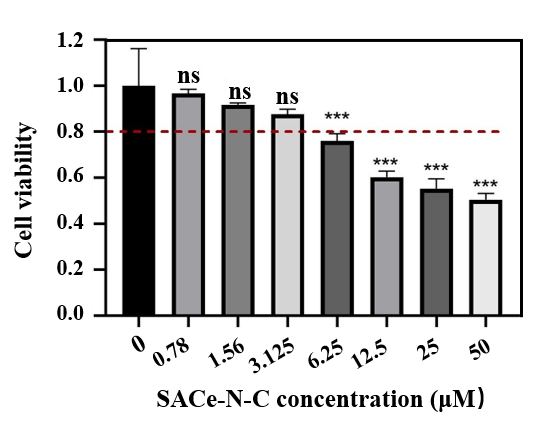


Figure S5. Cell viability assay.

We first studied the toxicity of the SACe-N_4_-C-(OH)_2_ nanozyme through the CCK8 cell viability kit. The results were shown in Figure S5, we selected the maximum concentration of SACe-N_4_-C-(OH)_2_ nanozyme was 3.125 μM for subsequent experimental treatment.


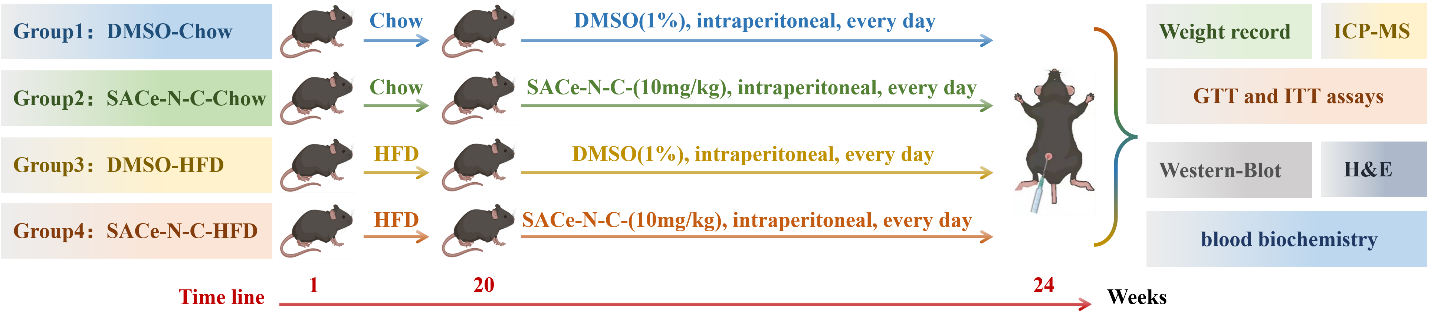


Figure S6. Treatment process of mouse.

The experimental design: after 20 weeks of the high-fat diet or chow diet induced hyperglycemia model, the experiment was divided into 4 groups: DMSO-Chow and DMSO-HFD group mice were given to 1%DMSO by intraperitoneal injection. The SACe-N_4_-C-(OH)_2_-Chow and SACe-N_4_-C-(OH)_2_-HFD group mice were given 10 mg/kg SACe-N_4_-C-(OH)_2_ nanozyme (dissolved in 1 %DMSO) by intraperitoneal injection for 4 weeks. Each group contained 6 mice. During treatment, DMSO-Chow and SACe-N_4_-C-(OH)_2_-Chow groups were given the chow diet, and the hyperglycemia model mice, such as DMSO-HFD and SACe-N_4_-C-(OH)_2_-HFD groups were fed on a high-fat diet.


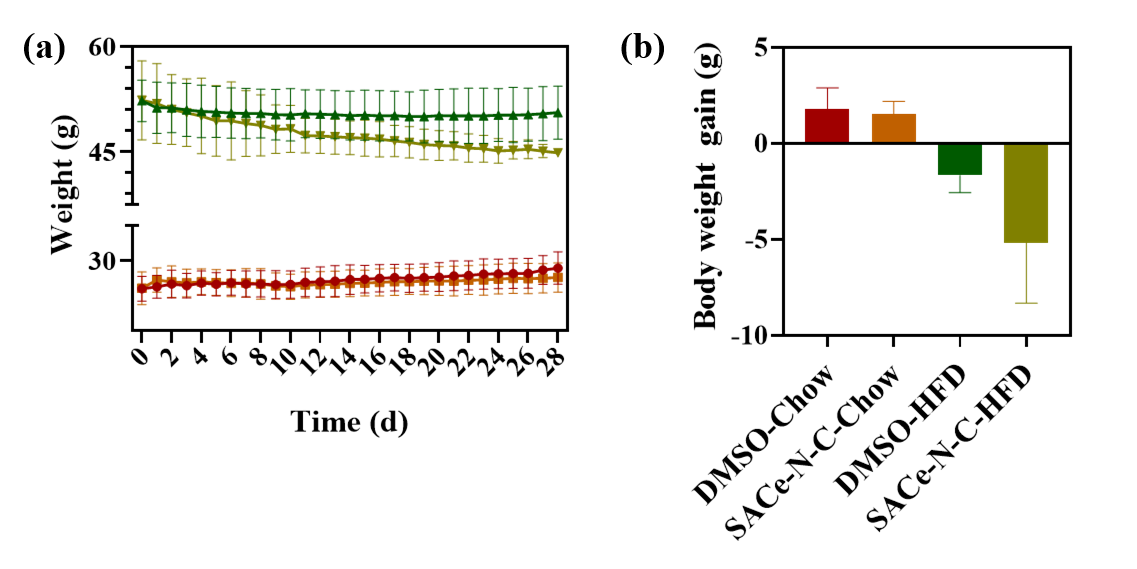


Figure S7. Body Weight

(a): the change of body weight; (b): body weight gain within 4 weeks.

After 20 weeks of the high-fat diet or chow diet induced hyperglycemia model, we recorded the weight of the mice daily for 4 weeks. It was found that the SACe-N_4_-C-(OH)_2_ nanozyme had no significant effect on the change of body weight of mice, nor on the change of body weight gain. It can be seen that SACe-N_4_-C-(OH)_2_ nanozyme had no effect on the body weight of normal mice and hyperglycemic mice.


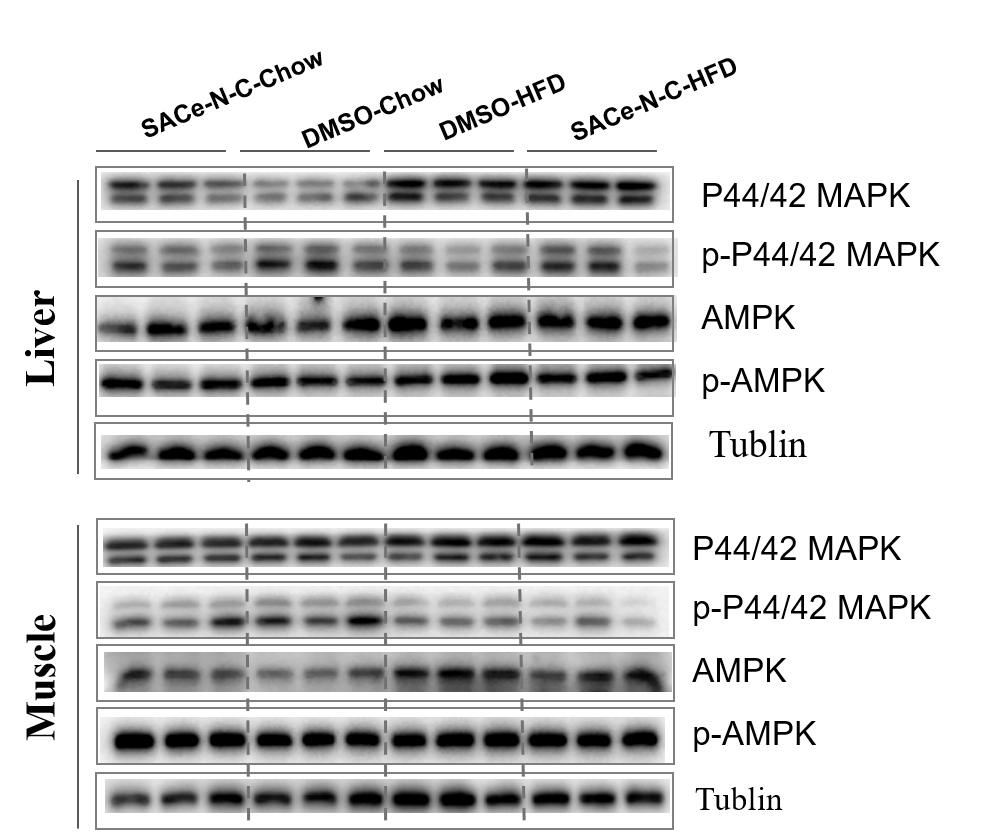


Figure S8. Western-blot in Liver and Muscle.

Liver and muscle tissues are the main organ tissues in the process of glucose metabolism, Therefore, we performed the western-blot analysis of AMPK levels in liver and muscle tissue after treated with SACe-N_4_-C-(OH)_2_ nanozyme.  It was found that SACe-N_4_-C-(OH)_2_ nanozyme had no effect on AMPK expression in liver and muscle tissues.


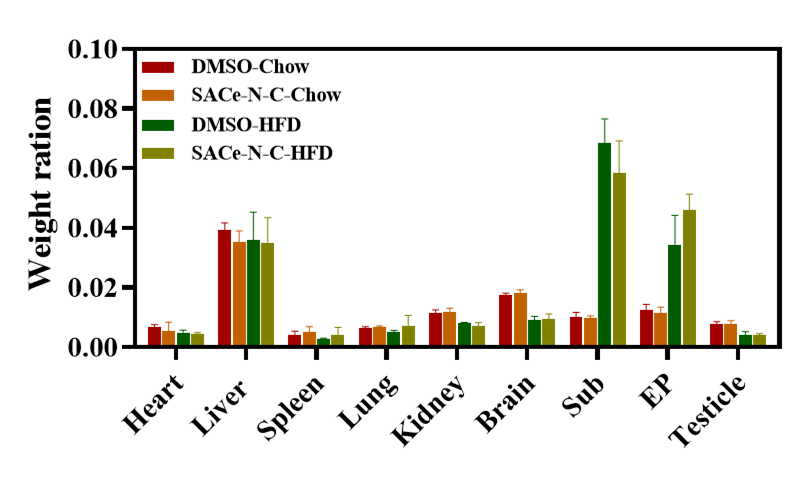


Figure S9. Weight ration.

After the mice were killed, we weighed the main organ tissues, such as heart, liver, spleen, lung, kidney, brain, sub, ep, and testicle. It was clearly found that SACe-N_4_-C-(OH)_2_ nanozyme had no significant effect on organ tissues in normal mice and hyperglycemic mice. It can be shown a degree of security.


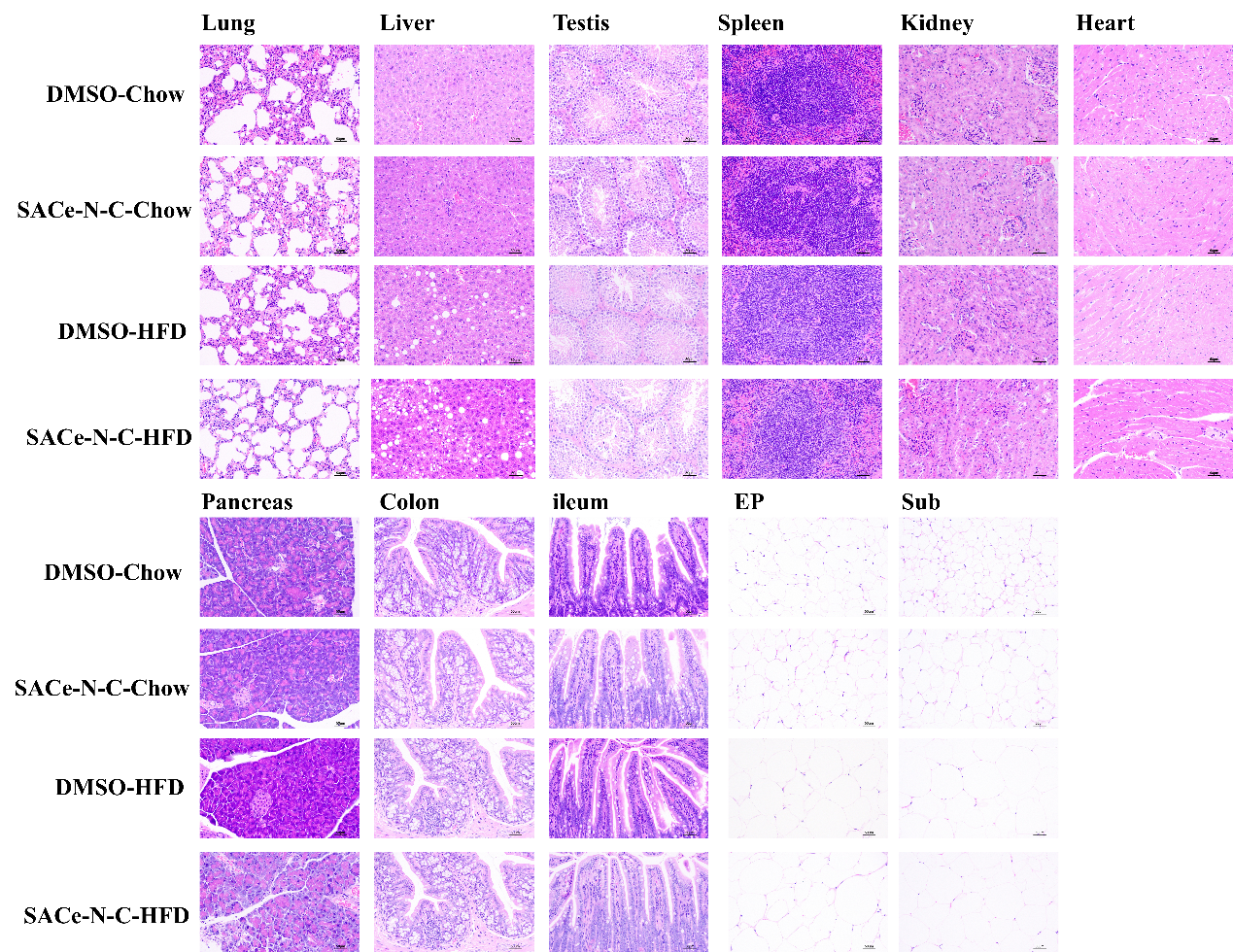


Figure S10. H&E staining of tissue sections from SACe-N_4_-C-(OH)_2_ nanozyme- or DMSO-treated C57BL/6J mice. Scale bar: 200 μm.

H&E staining was performed on major organs of mice, such as lung, liver, testis, spleen, kidney, pancreas, colon, ileum, ep and sub tissues. heart and liver.  From these results of pathological sections, it can be seen that SACe-N_4_-C-(OH)_2_ nanozyme had no obvious pathological damage to each organ tissue, and had a certain biological safety.


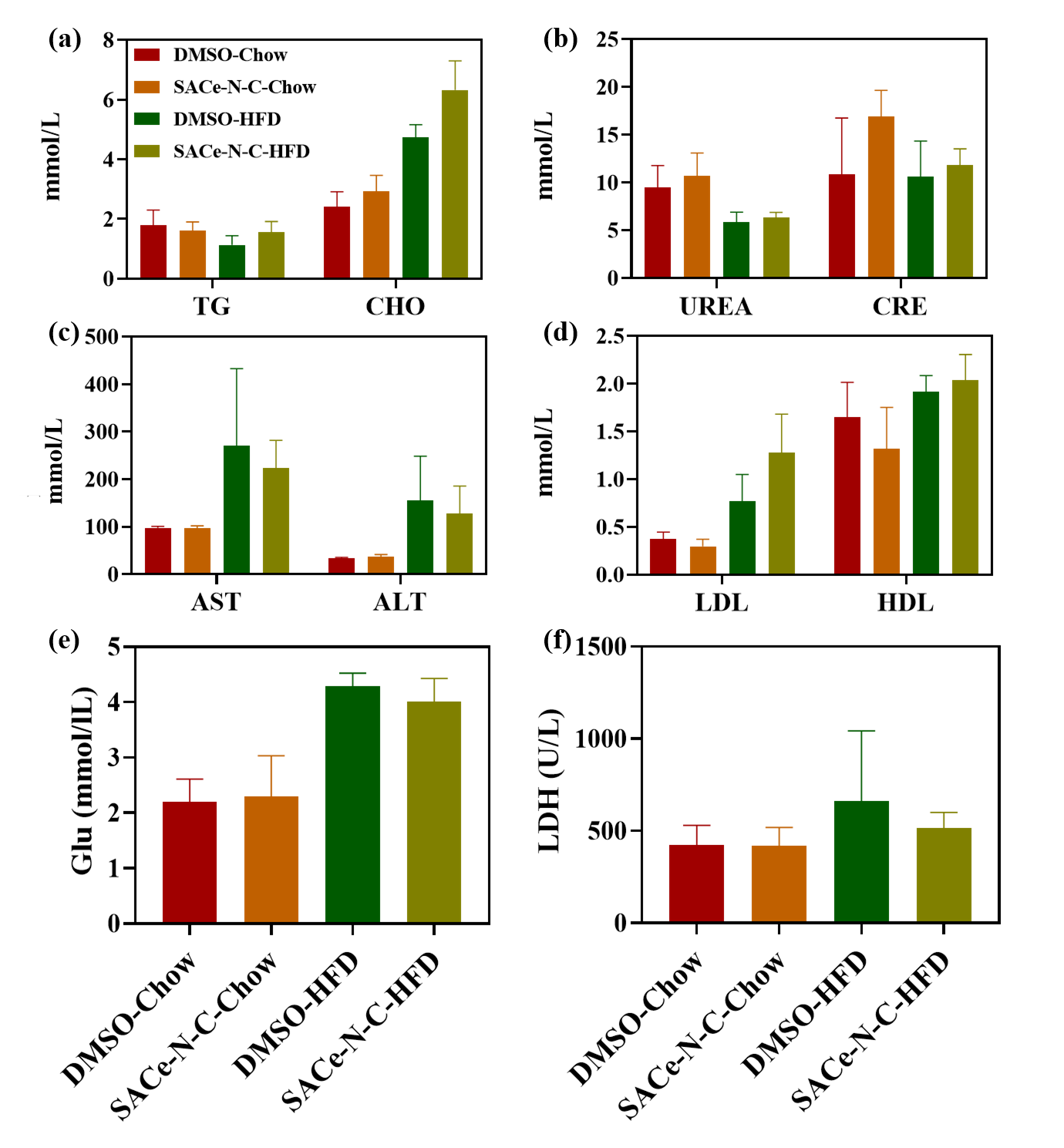


Figure S11. Complete blood panel analysis and blood biochemistry of control mice, and mice i.p. injected with SACe-N_4_-C-(OH)_2_ nanozyme at 4 weeks.

(a):Triacylglycerol (TG), Cholesterol (CHO); (b): Urea nitrogen (UREA), Creatinine (CRE);

(c): Aspartate aminotransferase (AST), Alanine amino transferase (ALT);

(d): Low density lipoprotein (LDL), High density lipoprotein (HDL);

(e): Glucose (Glu); (f): Lactic dehydrogenase (LDH).

By analyzing hematologic components, such as TG, CHO, UREA, CRE, AST, ALT, LDL, HDL, Glu, and LDH.  It can be easily observed that the treatment of SACe-N_4_-C-(OH)_2_ nanozyme had no obvious damage to the liver and kidney functions of normal mice and hyperglycemic mice, which indicates that it had biological safety.
